# The longevity landscape: mapping stakeholder priorities for healthy aging among high-income countries

**DOI:** 10.1186/s12889-025-25498-8

**Published:** 2025-12-19

**Authors:** Wasu Mekniran, Odile-Florence Giger, Elgar Fleisch, Tobias Kowatsch, Mia Jovanova

**Affiliations:** 1 Centre for Digital Health Interventions (CDHI), Department of Management, Technology, and Economics, ETH Zurich, Zurich, Switzerland; 2 CDHI, Institute of Technology Management, University of St. Gallen, St. Gallen, Switzerland; 3 CDHI, School of Medicine, University of St. Gallen, St. Gallen, Switzerland; 4 CDHI, Institute for Implementation Science in Health Care, University of Zurich, Zurich, Switzerland

**Keywords:** Healthy aging, Longevity, Public health systems, Value proposition, Health policy, Social determinants of health

## Abstract

**Objective:**

Global population aging presents significant economic and social challenges, requiring coordinated efforts to enhance healthy lifespans. However, little is known about how stakeholders prioritize healthy aging initiatives. We aimed to examine how a sample of stakeholders prioritize determinants of healthy aging, and what gaps or inconsistencies exist in stakeholder focus on these determinants?

**Methods:**

We conducted a first systematic analysis of the value propositions of 56 stakeholders, including governments, companies, research centers, opinion leaders, communities, and multilateral organizations, identified through Web of Science, PitchBook, and Crunchbase. Guided by the National Academy of Medicine’s All-of-Society framework, we assessed stakeholder emphasis on four determinants of healthy aging: public health systems, social factors, physical infrastructure, and work and education.

**Results:**

Public health system reform emerged as the most emphasized determinant, with significant focus on investing in geroscience and developing tailored primary care for older adults. In contrast, critical social and infrastructural factors—such as digital literacy, housing, transportation, financial security, and loneliness—received limited attention among stakeholders.

**Conclusions:**

These findings highlight the need for cross-sector partnerships to address these overlooked determinants and ensure a holistic approach to healthy aging. Future research should explore collaborative strategies to bridge these gaps to meet the diverse needs of aging populations.

**Supplementary Information:**

The online version contains supplementary material available at 10.1186/s12889-025-25498-8.

## Highlights

- The study systematically examines 56 stakeholders, highlighting the need for cross-sector partnerships to address neglected areas and adopt a holistic healthy aging approach.

- Identified stakeholders prioritize public health reform, focusing on geroscience and tailored primary care for older adults.

- Key social and infrastructural factors like loneliness, financial security, and housing are largely overlooked.

**Supplementary Information:**

The online version contains supplementary material available at 10.1186/s12889-025-25498-8.

## Background and theoretical motivation

The global demographic change toward an aging population and the rise of non-communicable diseases (NCDs) present a key challenge in today’s healthcare systems [1]. On an individual scale, older adults increasingly contend with NCDs, while at a societal level, healthcare systems face escalating costs. 74% (41 out of 55.4 million) of global deaths in 2019 were attributed to NCDs [1]. In parallel, global healthcare expenditure is projected to surge from $9.1 trillion in 2020 to $11 trillion by 2026 [2]. Global aging is a major driver of rising healthcare costs, with expenditures for older adults being three to five times higher than those for younger individuals [3, 4]. By 2050, the number of people aged 60 and older is expected to double, reaching 2.1 billion [5]. Promoting healthy aging—defined as maintaining physical, mental, and social well-being in older age and enhancing quality of life—is a critical first step to address the social, economic, and healthcare challenges associated with this demographic shift [6].

In recognition of this urgent issue, the World Health Organization (WHO) designated 2021–2030 as the Decade of Healthy Aging [7]. Similarly, the National Academy of Medicine (NAM) convened a multidisciplinary commission to develop “The Global Roadmap for Healthy Longevity”,a framework to address the economic and health challenges posed by aging populations [8]. Central to this framework is an ‘All-of-society’ approach, which advocates for collaboration among diverse stakeholders—government agencies, multilateral organizations, companies, research institutions, local communities, and individual opinion leaders. Drawing on this perspective, Wong, Fried and Dzau [9] proposed key focus areas to translate the health and economic challenges of global population aging into new opportunities for societies, including increased economic contributions from older adults through extended workforce participation and enhanced social engagement.

Public health systems struggle to address the needs of older populations, hindered by fragmented primary care and limited access to long-term care. Globally, around 60% of older adults lack access to essential long-term care services [10]. In response, research has called for systemic reforms that prioritize equitable access to primary and long-term care services and enable culturally sensitive and person-centered care [11]. Equally critical are investments in social and physical infrastructure determinants that enable healthier lifestyles for older adults [12]. Many older adults face barriers to healthy aging, including limited access to affordable housing [13], and nutritious food [14, 15].

Social initiatives should also focus on combating loneliness and fostering community by strengthening social networks among older adults [16–18]. Ageism and workplace exclusion often diminish cognitive engagement and social interactions and negatively impact well-being [19, 20]. Creating opportunities for older adults to work and learn can sustain cognitive health, and improve overall quality of life [21, 22]. Additionally, supporting workforce participation and promoting lifelong learning for older adults are essential to enhancing engagement of older adults [23, 24]. Furthermore, investing in geroscience research is crucial to understanding biological aging and developing targeted interventions. Increased investment in tailored educational and behavioral programs is also necessary to promote adaptability and resilience among older adults [25]. By integrating these multi-sector priorities—reforming public health systems, addressing social determinants of health, stakeholders can together enhance population health across the lifespan. These coordinated efforts are vital to realizing the "longevity dividend," the societal benefits of longer, healthier lives, including reduced healthcare costs from lower NCD prevalence and increased social capital through active older adult participation in community volunteering [9]. For an overview of the key determinants of healthy aging within these categories, refer to Table 1.

**Table 1:** Determinants of healthy aging (adapted from [9])

Social Determinants	Physical Infrastructure	Work, Science & Education	Health Systems
Tackling ageism	Housing and public spaces	Work environments for older adults	Investing in geroscience
Building networks to tap the prosocial goals and strengths of older people	Public transportation	Policies encouraging older adults to remain in the workforce	Quality primary care
Reducing loneliness across the life course	Access to digital technology	Volunteerism	Public health interventions
Establishing financial security in older age	Mitigating environmental emergencies and reducing air pollution	Redesigning education systems to support lifelong learning, training	Investments in person centered care
Ensuring digital literacy	User-centered design co-created with older adults	Investing in the science of learning and training for middle-aged and older adults	Culturally sensitive and equitable long-term care

### The current study

While Wong, Fried, and Dzau [9] outlined evidence-based priorities for addressing global aging, a key gap persists in understanding how stakeholders prioritize key healthy aging determinants. To date, no study has explored whether, and to what extent, stakeholders focus on these four key determinants, namely reforming public health systems, addressing social determinants of health, improving physical infrastructure, and expanding opportunities in work, science, and education. Existing research has centered on a single stakeholder perspective, such as the researcher community focus on geroscience [26]. However, understanding different stakeholder priorities across the landscape is crucial to identifying synergies and gaps between sectors [27]. By mapping these priorities, it becomes possible to pinpoint areas of alignment for collaboration and neglected domains requiring additional attention and resources [28–30]. Such insights are essential for designing strategies for healthy aging and ensuring that no determinant category—whether public health systems, infrastructure, or opportunities in work—is overlooked in global aging responses [31, 32].

Thus, building on the *All-of-Society* framework [8] and extending the work by Wong, Fried, and Dzau [9], this study examines how identified stakeholders prioritize healthy aging determinants. Drawing on public health and management research [28, 33], we map the value propositions—defined as the distinct benefits stakeholders seek to provide through product or service, such as enhanced quality of life or cost savings—among stakeholders in the longevity landscape to examine the following two research questions:

1. How prevalent are healthy aging determinant categories in the value propositions of relevant stakeholders?
2. How do stakeholder value propositions align or differ in their emphasis on specific healthy aging determinants?

## Methods

We conducted a descriptive thematic analysis [34] to identify healthy aging determinants in the value propositions among stakeholders in the longevity landscape. This methodological approach was chosen because it facilitates an in-depth exploration of perspectives of diverse stakeholders, a comparative analysis of different stakeholders, and allows us to identify thematic overlap and gaps in stakeholder value propositions [28, 35].

### Search strategy and data collection

Building on prior similar descriptive work in the public policy and health promotion [31, 32], we used a combination of databases [36–38] to capture both academic and industry stakeholders. Specifically, we used Web of Science (Clarivate) for its capability to filter by institutional affiliation and to rank organizations by number of publications, which enabled a transparent identification of influential academic institutions. To complement this, we conducted searches in April 2024, using PitchBook [39], and Crunchbase [40], databases which offer industry data on private companies and key opinion leaders, to identify stakeholders—government organizations, multilateral organizations, researcher centers, private companies, local communities, and opinion leaders in the healthy longevity landscape [41]. Other databases (e.g., Ovid Medline, CINAHL, Google Scholar) were not selected as they lack the standardized, reproducible institution-level filtering and ranking needed for our analysis [42]. No protocol was registered for this review. See ‘Table 2’ for details on our search terms and eligibility criteria for stakeholder inclusion. To be considered eligible, stakeholders needed to meet two predefined inclusion criteria: (a) involvement in initiatives related to healthy longevity and (b) objectives explicitly aligned with promoting healthy aging or longevity [7, 9]. We further adopted stakeholder-specific eligibility criteria to capture “key” stakeholders who are most likely to drive global healthy longevity initiatives [43]. Specifically, governmental agencies, multilateral organizations, and research centers were selected based on ranking the total funding devoted to healthy longevity scientific research initiatives [44, 45] with stakeholders like the National Institute on Aging and Mayo Clinic included for their direct focus on healthy aging research and programs, ranking by a number of publications. Private companies were selected based on the total funding amount allocated to longevity-focused healthy aging enterprises [46–50], though organizations like the National Science Foundation and WebMD were not captured in our systematic search, as their primary focus lies outside longevity-specific interventions. Local communities were selected based on the funding devoted to community initiatives aimed at promoting healthy aging. Individuals, or opinion leaders, were selected based on their level of individual involvement in key global longevity events and congresses, such as participation in speaking events [51]. We focused our analysis on the key stakeholders by selecting the first ten entries that met the criteria for each stakeholder group to ensure a manageable and representative example for in-depth review. Two coders reviewed each stakeholder, and a third coder resolved any conflicts between their assessments [52].

**Table 2:** Search strategy for global stakeholders active in the healthy aging landscape

Stakeholder	Database	Criteria	Search Terms	Examples
(1) Government agencies	Web of Science	Total funding devoted to healthy longevity scientific research	Topic: ("Healthy Ageing" OR "Healthy Longevity")	UN, WHO, EU
(2) Multilateral organizations	Web of Science	Total funding devoted to healthy longevity scientific research	Topic: ("Healthy Ageing" OR "Healthy Longevity")	Wellcome Trust, AGE-WELL
(3) Research centers	Web of Science	Total funding devoted to healthy longevity scientific research	Topic: ("Healthy Ageing" OR "Healthy Longevity")	MIT AgeLab, Buck Institute for Research on Aging
(4) Private companies	Crunchbase/PitchBook	Total funding amount devoted to longevity- programs, communities, or private enterprises	Keywords: Healthy Longevity OR Healthcare OR Biological age; Industry function: Health care	Altos Labs, Devoted Health, Grail, WeDoctor, MGI Tech
(5) Communities	Crunchbase/PitchBook	Total funding amount devoted to longevity- programs, communities, or private enterprises	Keywords: Healthy Longevity OR Healthcare OR Biological age; Industry function: Communities	Cedars Sinai, MaineHealth, Heartland Family Service
(6) Opinion leaders	Crunchbase/PitchBook	Individual participation in the highest number of global healthy longevity events	Event function: Longevity Leaders World Congress OR International Longevity Policy and Governance Summit	David Sinclair, Aubrey de Grey, Jim Mellon, James Peyer

### Value proposition extraction, coding and analysis

For each eligible stakeholder, two independent coders extracted value propositions from publicly available sources, including scientific publications (Web of Science), financial data (Pitchbook and Crunchbase, and organizational websites. To address our two key research questions, we conducted a descriptive thematic analysis to identify stakeholder priorities, building on identified value propositions to categorize data into themes. First, to examine how prominently healthy aging determinants appear in the value propositions of relevant stakeholders in the global longevity, we described the prevalence of themes across four determinants (health systems, social determinants, physical infrastructure, work, science, and education), While all are connected to the broad concept of social determinants of health, we intentionally disaggregated them to highlight their distinct roles in shaping healthy aging and the different policy levers and stakeholders involved. For instance, physical infrastructure (e.g., housing, transportation, digital technology) depends on urban planning and technology sectors, whereas work and education require labor market and lifelong learning policies. Similarly, health systems interventions draw on public health and geroscience, while core social determinants such as ageism, social networks, and financial security rely on social policy and community engagement [8]. The extracted value propositions were systematically coded based on 20 healthy aging determinants spanning four categories: health systems, social determinants, physical infrastructure, work, science, and education, as outlined by Wong, Fried & Dzau [9] and summarized in Table 1. To highlight the prevalence patterns, we calculated the percentage coverage of each healthy aging determinant relative to the total references to all of the healthy aging determinant themes for each stakeholder group. For a given category ${C}_{i}$ and stakeholder group $S$, the percentage coverage was defined as:

$$\begin{aligned}&{\text{Percentage Coverage}}_{{c}_{i},S}=\\&\left(\frac{\text{Count of references} to {C}_{i} \text{by} S}{\text{Total count of references to all categories by }S}\right)\times 100\end{aligned}$$

To next identify how stakeholders align or differ in their emphasis on healthy aging determinants, we analyzed the degree to different stakeholder value propositions referenced similar or different healthy aging determinants, distinguishing between widely shared and emphasized stakeholder priorities. Finally, figures are generated using R v4.4 with the ggplot2 v3.3 with ggalluvial package. In addition to value propositions and determinant themes, we extracted contextual data on stakeholder type, country or region, and funding stage. Where information was incomplete, organizational websites were cross-checked to verify details.

## Results

### Descriptives

Our final list included *N* = *56* stakeholders (research centers = 10; private companies = 10; communities = 10; opinion leaders = 10; governmental agencies = 7; multilateral organizations = 9). Of the 60 stakeholders initially identified, four were excluded as they did not demonstrate a direct focus on healthy longevity or had insufficient publicly available information for classification. See Table S1 in the supplements for descriptive details, such as geographic coverage of the included stakeholders. The average number of longevity-focused peer-reviewed publications funded by governmental agencies was 126 (SD = 123), multilateral organizations 31 (*SD* = 41), and research centers 44(*SD* = 89, range = 4–308). In comparison, private companies focused on longevity and healthy aging reported an average funding amount of $1,558,430,283 (*SD* = $671,679,823, range = $792,722,908–$3,000,000,000), while local community initiatives dedicated to healthy aging received an average of $16,319,887 (*SD* = $18,010,738, range = $5,000,000–$63,310,000). Additionally, opinion leaders participated in an average of 10 (*SD* = 2.5) global longevity events.

Collapsing the value propositions across all stakeholders, the most prevalent healthy aging determinants included public health system reform (38%), followed by developing new work, education, and science opportunities (27%); addressing social determinants (19%), and improving physical infrastructure (16%). The five most represented healthy aging determinants (collapsed across all stakeholders) included: investing in geroscience (9.9%); developing public health interventions for healthy aging (8.16%); promoting culturally tailored long-term care (7.48%); investing in science of learning and training for middle-aged and older adults (7.48%); and improving quality primary care (7.14%), accounting for 40.16% of the prevalence all healthy aging determinants. The five least represented healthy aging determinants (collapsed across all stakeholders) included: promoting initiatives to reduce loneliness (2.04%); mitigating air pollution and environmental changes (2.04%); improving access to public spaces (1.36%); access to digital literacy (1.36%) and access to public transportation (1.02%), accounting for 7.82% of all healthy aging determinants. See Table 3 for all healthy aging determinants across stakeholders.

**Table 3:** Prevalence of healthy aging determinants among global stakeholders (*N* = *56*)

Determinants of Healthy Aging	Count (n)	Proportion (%)	Diversity of stakeholders	Stakeholder Categories
Health systems: Geroscience	29	9.80%	6	Governing agencies, Companies, Research centers, Opinion Leaders, Local communities, Multilateral
Social determinants: Social Networks	24	8.16%	3	Governing agencies, Research centers, Opinion Leaders, Local communities, Multilateral
Health systems: Public Health Interventions	22	7.48%	5	Governing agencies, Research centers, Multilateral
Health systems: Culturally Sensitive Care	22	7.48%	5	Governing agencies, Research centers, Opinion Leaders, Local communities, Multilateral
Work, science & education: Science of learning	21	7.14%	6	Governing agencies, Research centers, Opinion Leaders, Local communities, Multilateral
Social determinants: Ageism	21	7.14%	5	Governing agencies, Research centers, Opinion Leaders, Local communities, Multilateral
Work, science & education: Tailored work environments	20	6.8%	5	Governing agencies, Companies, Research centers, Local communities, Multilateral
Health systems: Quality primary care	18	6.12%	5	Governing agencies, Companies, Research centers, Opinion Leaders, Local communities, Multilateral
Physical infrastructure: Digital Technology	18	6.12%	4	Governing agencies, Companies, Research centers, Local communities, Multilateral
Health systems: Person-centered care	17	5.78%	5	Governing agencies, Companies, Research centers, Local communities, Multilateral
Work, science & education: Work policies	17	5.78%	4	Governing agencies, Research centers, Local communities, Multilateral
Physical infrastructure: User centered-design	16	5.44%	3	Governing agencies, Companies, Research centers
Work, science & education: Lifelong Training	10	3.4%	4	Governing agencies, Research centers, Local communities, Multilateral
Work, science & education: Volunteerism	9	3.06%	4	Governing agencies, Research centers, Local communities, Multilateral
Social determinants: Loneliness	7	2.38%	3	Governing agencies, Research centers, Local communities
Physical infrastructure: Air pollution	6	2.04%	2	Governing agencies, Multilateral
Social determinants: Financial security	6	2.04%	2	Local communities, Multilateral
Physical infrastructure: Housing & public spaces	4	1.36%	2	Governing agencies, Research centers
Social determinants: digital literacy	4	1.36%	2	Governing agencies, Research centers
Physical infrastructure: Public transportation	3	1.02%	1	Governing agencies

### (RQ1) prevalence of healthy aging determinants across stakeholder value propositions

**Fig. 1:**
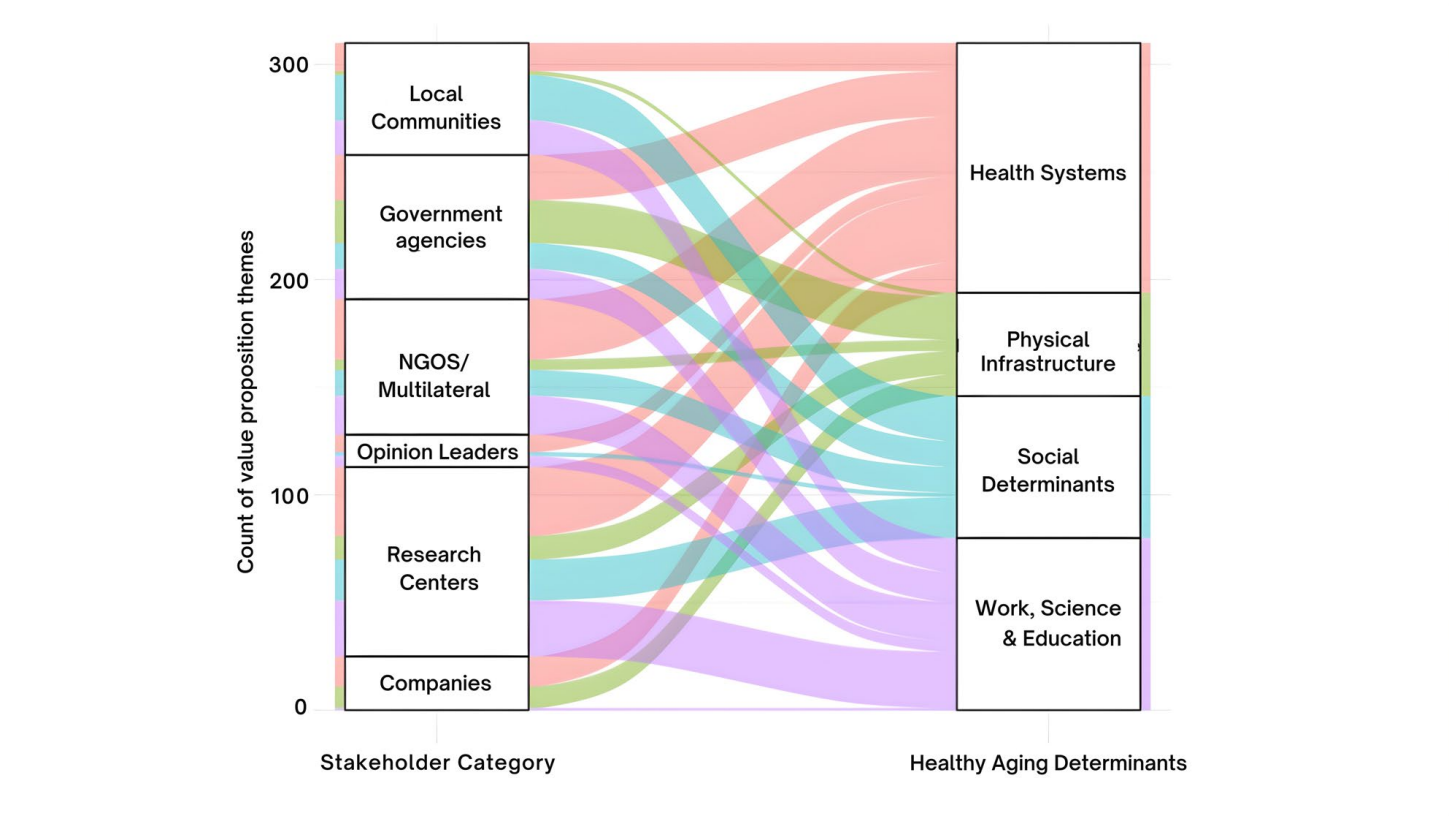
Patterns of Healthy Aging Determinants Across Stakeholder Value Propositions. Distribution of healthy aging determinant categories across stakeholders’ value propositions, with ‘public health systems’ emerging the most emphasized determinant across all stakeholder groups and the only category referenced by all six groups

The prevalence of the healthy aging determinants categories across stakeholders’ value propositions is illustrated in Fig. 1. This breakdown reveals a high degree of shared focus on public health system reform, but also shows wide variation in the emphasis placed on the other three categories: ‘social determinants’, ‘work, education, and science’, and ‘physical infrastructure’.

The most emphasized category among governing agencies was public health system reform (33.9%), followed by improving physical infrastructure (32.3%), investing in work, education, and science opportunities (22.6%), with addressing social determinants receiving the least attention (11.3%). For multilateral organizations, public health system reform was similarly the top priority (44.4%), followed by investing in work, education, and science (28.6%), while addressing social determinants (19%) and improving physical infrastructure (7.9%) were less frequently emphasized. Private companies strongly focused on public health system reform (56.5%) followed by improving physical infrastructure (39.1%), and investing in work, education, and science opportunities (4.35%). In turn, addressing social determinants received no mention. For research centers, public health system reform was the leading category (37.2%), followed by investing in work, education, and science (32.1%). Addressing social determinants (16.7%) and improving physical infrastructure (14.1%) were less prominent. Among opinion leaders, the primary focus was on public health systems reform (53.3%), with secondary emphasis on investing in work, education, and science (33.3%), and addressing social determinants (13.3%), and with no mention of social determinants. In contrast to other stakeholders, local communities placed the most emphasized category on addressing social determinants (39.6%), followed by investing in work, education, and science (30.2%), public health system reform (26.4%), and improving physical infrastructure (3.8%).

### (RQ2) overlap and gaps in healthy aging determinants across stakeholder value propositions

**Fig. 2:**
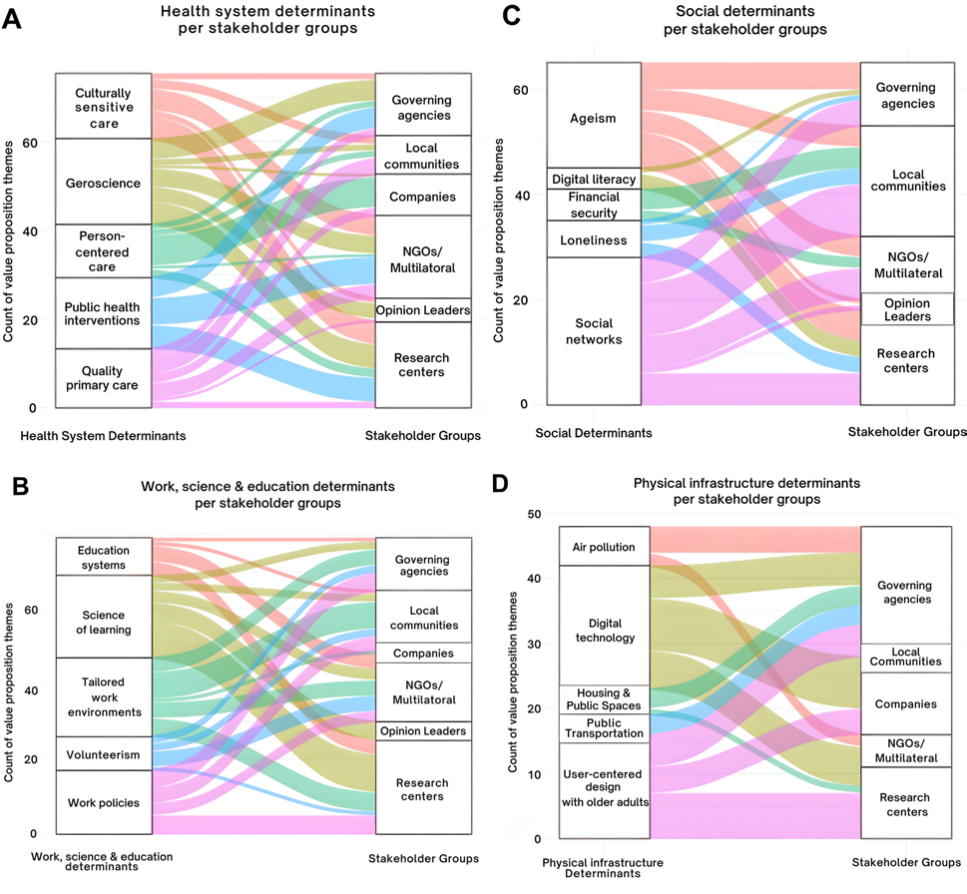
Stakeholder Priorities in Healthy Aging Determinants Show Wide Variation. Healthy aging determinants referenced across stakeholder value propositions, divided into four panels: social determinants (**A**), physical infrastructure (**B**), public health systems (**C**), and work, education, and science (**D**). In panels (**B**) and (**C**), private companies are excluded from the stakeholder categories as they did not reference any 'social determinants' or 'physical infrastructure' themes related to healthy aging

We observed a consistent emphasis on the broader category of ‘public health systems’ across all stakeholder groups (Fig. 1). However, the prioritization of specific determinants within this category varied substantially. For example, the determinants ‘investing in geroscience’ and ‘quality primary care’ were referenced by all six stakeholder groups, but their prevalence ranged widely—from 7.6% to 62.5% for ‘investing in geroscience’ (M = 27.8%, SD = 19.3%) and from 6.9% to 50% for ‘quality primary care’ (M = 20.7%, SD = 15.3%) (Fig. 2A). Other determinants, such as ‘improving culturally sensitive long-term care’ (M = 21.7%, SD = 7.1%, range = 9.5%–27.6%) and ‘person-centered care’ (M = 21.4%, SD = 27.0%, range = 3.6%–69.2%), were mentioned by five stakeholder groups, showing notable variability in prevalence. In contrast, determinants like ‘developing public health interventions’ were referenced by only three stakeholder groups, with a narrower range (M = 31.0%, SD = 3.0%, range = 27.6%–33.3%). This variation underscores a shared prioritization of public health systems as a whole, while revealing distinct sub-areas of focus among different stakeholder groups (Fig. 2A).

For social and physical infrastructure determinants, ‘building social networks to tap the prosocial goals and strengths of older people’ was referenced by five out of six stakeholder groups, with a prevalence range of 7.7%−50% (*M* = 38.8%, *SD* = 20.8%). However, other determinants, such as ‘reducing loneliness’ (*M* = 13.1%, *SD* = 14.29%; range = 8.33%−16.67%) and ‘user-centered design co-created with older adults,’ 19.5%−63.6% (*M* = 42.9%, *SD* = 40%) were mentioned by only three stakeholder groups (Fig. 2C). Additional determinants under public infrastructure, including ‘financial security,’ 16.7%−19.1% (*M* = 17.9%, *SD* = 17.7%), ‘digital literacy,’ 8.3%−16.7% (*M* = 12.5%, *SD* = 12.5%), ‘air pollution,’ 20%−40% (*M* = 30%, *SD* = 14.4%) and ‘housing and public spaces,’ 9.1%−15% (*M* = 12.1%, *SD* = 4.18%) were referenced by only two stakeholder groups. Notably, ‘access to public transportation’ was mentioned by just one group (governing agencies), accounting for 15% of the prevalence proportion (Fig. 2B). Overall, these findings demonstrate that while there is alignment on the broader focus of public health systems, stakeholders differ widely in the specific aspects they prioritize. Social and public infrastructure determinants received the least overlap and representation among stakeholders, revealing potential gaps in social and structural determinant emphasis across stakeholder groups, visualized in Fig. 2 and summarized in Table 3.

## Discussion

This study provides a systematic analysis of how a sample of stakeholders prioritizes healthy aging determinants. We uncovered patterns of both alignment and gaps, with a shared emphasis on reforming public health systems towards the needs of older adults. However, while reforming public health systems were a unifying focus, emerging as the top healthy aging determinant category for five out of six stakeholder groups, stakeholders differed in how they operationalized this goal. Multilateral organizations prioritized developing public health interventions to improve population-level health outcomes. Private companies, on the other hand, focused on developing person-centered solutions like digital health platforms to empower individuals in managing their health, such as Hinge Health, a digital platform for musculoskeletal care, and Lyra Health, a digital platform for mental health. Local communities concentrated on improving access to high-quality primary care tailored to older adults, particularly in underserved areas. Governing agencies directed their efforts toward funding geroscience research and large-scale public health initiatives, while opinion leaders emphasized the importance of translational geroscience research to bridge the gap between scientific discoveries and practical applications.

Despite a shared focus on improving health systems, other important determinants of healthy aging, such as social and physical infrastructure, were underrepresented. For instance, while local communities emphasized the need to reduce loneliness and improve social connections among older adults, these issues received less attention from other stakeholders. Similarly, factors such as access to housing, transportation, and the environment—were given limited focus, despite their well-established significance in the literature [12]. This gap points to a potential misalignment between theoretical frameworks advocating for a holistic approach to aging and the practical priorities or immediate concerns of stakeholders.

This study builds on the work of Wong, Fried, and Dzau [9] by providing a stakeholder-specific analysis of the determinants of healthy aging. While previous research has advocated for an "All-of-Society" approach to addressing global aging challenges, this study operationalizes that concept by systematically mapping how multiple stakeholders prioritize these determinants. Unlike earlier work that typically takes a singular perspective, for example those of geoscience researchers [26] this study captures a diverse range of stakeholder viewpoints, highlighting shared priorities, such as the emphasis on public health systems, and gaps, particularly in addressing social and physical infrastructure determinants.

The results are composed predominantly of high-income stakeholders, which contrasts with the global challenge of healthy aging, where LMIC governments, communities, and organizations are central, as 80% of older adults are expected to live in LMICs by 2050 [53]. Guided by the UN Decade of Healthy Ageing (2021–2030) and MIPAA [54], efforts focus on supportive environments, NCD prevention, economic security, and social engagement. Examples include the Caribbean’s CARICOM Charter, fostering age-friendly communities via coordinated health policies across 15 states [55], and Barbados’ National Policy, integrating primary care for over 20,000 elders. In Africa, Ghana’s policy supports 50,000 caregivers with stipends and training [56], while Kenya’s model establishes 47 geriatric clinics since 2018. Asia’s India policy offers free screenings to 10 million seniors [57], and Latin America’s Brazil policy provides home care to 30,000 elderly since 2015 [58], with the Commonwealth integrating early NCD screening across 56 nations [59]. Interventions like India’s Rashtriya Vayoshri Yojana aid 500,000 elders with devices since 2017, and Barbados’ programs engage 15,000 in NCD risk reduction, aligning with equity goals despite challenges of resource shortages and limited elder input. Integrating these strategies enhances cross-regional learning for improved aging outcomes. While this is the first study, to our knowledge, to systematically map stakeholders' priorities among a sample of stakeholders in the longevity landscape, it has several limitations. First, the geographical concentration may limit the generalizability, as 29 of 56 stakeholders (51.79%) were based in the United States, with the remainder distributed as follows: Europe (17, 30.36%), Asia (6, 10.71%), Canada (2, 3.57%), and others (2, 3.57%). The study also does not account for changes in stakeholders' priorities over time, potentially missing evolving trends of external factors. Additionally, the reliance on publicly available data from platforms like Web of Science, PitchBook, and Crunchbase, which prioritize certain academic and available industry sources, may exclude certain stakeholders, leading to a bias toward well-established organizations, and these exclusions may underrepresent grassroots organizations and diverse perspectives. It is also important to note that this analysis reflects stakeholders' stated value propositions, not the actual outcomes of their initiatives. To address these limitations, future research may incorporate a broader geographical scope, especially LMIC, and utilize a more diverse data collection approach, including interviews or case studies. This could help validate stakeholders' stated priorities by comparing them to actual health system outcomes and identifying discrepancies between intended and real-world impacts, especially in areas such as healthcare access, financing, and service quality.

The findings of this study suggest actionable steps for fostering a more integrated approach to healthy aging. To address gaps in social and physical infrastructure, stakeholders could create cross-sector working groups that bring together local communities, private companies, and government agencies [60]. For instance, private companies with a focus on healthy aging offerings could expand their service models to include interventions targeting social determinants, such as loneliness reduction [16] or digital literacy [15]. Research institutions could collaborate with private companies to evaluate the interventions aimed at promoting healthy aging. At the same time, governing agencies and multilateral organizations can play a key role by funding evidence-based interventions and ensuring their scalability and sustainability. These efforts could target social and physical infrastructure determinants, such as improving public transportation and reducing air pollution. Initiatives like community-driven housing projects or digital literacy workshops for older populations could be supported through grants or matching funds, fostering local engagement and tailored solutions to community needs.

Public–private collaborations can leverage the strengths of diverse stakeholders. For example, partnerships between the Centers for Medicare & Medicaid Services (CMS) and digital health companies like Livongo and Lark—known for using AI-driven health coaching and real-time health data—illustrate how innovative approaches can improve outcomes for chronic conditions like diabetes. By fostering similar collaborations and aligning efforts, stakeholders can develop holistic strategies to address both social and physical determinants of healthy aging.

## Conclusions

Overall, this study systematically examined how a sample of stakeholders prioritizes healthy aging determinants, uncovering areas of alignment and critical gaps. While public health systems emerged as a shared priority, the limited focus on social and physical infrastructure underscores the need for more holistic strategies to address healthy aging. These findings highlight the importance of cross-sector collaboration, targeted resource allocation, and innovative partnerships to address aging. Building on the Global Roadmap for Healthy Longevity, this study moves beyond theoretical frameworks by providing a first descriptive analysis of stakeholder priorities in the global landscape. Future research should focus on developing and testing collaborative, multi-sector strategies to address social and physical determinants of healthy aging.

## Supplementary Information

Supplementary Material 1.

## Data Availability

The datasets generated and/or analyzed during the current study are available from the corresponding author upon reasonable request.

## References

[1] WHO. Non communicable diseases Key Facts. 2023. Available at: https://www.who.int/news-room/fact-sheets/detail/noncommunicable-diseases. Accessed: 12 March 2024.

[2] IHME. Financing Global Health. University of Washington. 2023. Available at: https://vizhub.healthdata.org/fgh/.

[3] Bakx P, O’Donnell O, van Doorslaer E. Spending on Health Care in the Netherlands: Not Going So Dutch. Fiscal Studies. 2016;37(3–4):593–625. Available at: 10.1111/j.1475-5890.2016.12114.

[4] OECD. Expenditure by disease, age and gender FOCUS ON Health Spending. 2016. Available at: https://www.oecd.org/els/health-systems/Expenditure-by-disease-age-and-gender-FOCUS-April2016.pdf. Accessed: 15 October 2023.

[5] United Nations. The Report on the World Social Situation 2018: Promoting Inclusion Through Social Protection. UN (Report on the World Social Situation). 2018. Available at: 10.18356/5ef37a49-en.

[6] Bautmans I, et al. WHO working definition of vitality capacity for healthy longevity monitoring. Lancet Healthy Longev. 2022;3(11):e789–96. 10.1016/S2666-7568(22)00200-8. 36356628 10.1016/S2666-7568(22)00200-8 PMC9640935

[7] WHO. *Decade of healthy ageing: baseline report*. Geneva: World Health Organization. 2020. Available at: https://apps.who.int/iris/handle/10665/338677. Accessed: 12 September 2022.

[8] National Academy of Medicine. Global roadmap for healthy longevity. Washington, D.C.: National Academies Press; 2022. p. 26144. 10.17226/26144. 36479752

[9] Wong JEL, Fried LP, Dzau VJ. The global roadmap for healthy longevity: United States of America National Academy of Medicine consensus study report, 2022. J Econ Ageing. 2023;24:100421. 10.1016/j.jeoa.2022.100421.

[10] The Lancet Healthy Longevity. Care for ageing populations globally. The Lancet Healthy Longevity. 2021;2(4):e180. Available at: 10.1016/S2666-7568(21)00064-7. 10.1016/S2666-7568(21)00064-7 PMC8529576 34697611

[11] Kogan AC, Wilber K, Mosqueda L. Person‐Centered Care for Older Adults with Chronic Conditions and Functional Impairment: A Systematic Literature Review. J Am Geriatr Soc. 2016;64(1). Available at: 10.1111/jgs.13873. 10.1111/jgs.13873 26626408

[12] Kaeberlein M, Rabinovitch PS, Martin GM. Healthy aging: the ultimate preventative medicine. Science. 2015;350(6265):1191–3. 10.1126/science.aad3267. 26785476 10.1126/science.aad3267 PMC4793924

[13] Alley DE, et al. Material resources and population health: disadvantages in health care, housing, and food among adults over 50 years of age. Am J Public Health. 2009;99(S3):S693–701. 10.2105/AJPH.2009.161877. 19890175 10.2105/AJPH.2009.161877 PMC2774171

[14] Pooler JA, et al. Food insecurity: a key social determinant of health for older adults. J Am Geriatr Soc. 2019;67(3):421–4. 10.1111/jgs.15736. 30586154 10.1111/jgs.15736 PMC6816803

[15] Baluk KW, Detlor B, La Rose T, Alfaro-Laganse C. Exploring the digital literacy needs and training preferences of older adults living in affordable housing. J Technol Hum Serv. 2023;41(3):203–29. 10.1080/15228835.2023.2239310.

[16] Bower M, et al. The social determinants of loneliness during COVID-19: personal, community, and societal predictors and implications for treatment. Behav Chang. 2023;40(1):1–10. 10.1017/bec.2023.3.

[17] Chidambaram S, et al. An introduction to digital determinants of health. PLoS Digit Health. 2024;3(1):e0000346. 10.1371/journal.pdig.0000346. 38175828 10.1371/journal.pdig.0000346 PMC10766177

[18] Kondo K (ed.). Social Determinants of Health in Non-communicable Diseases: Case Studies from Japan. Singapore: Springer (Springer Series on Epidemiology and Public Health). 2020. Available at: 10.1007/978-981-15-1831-7.

[19] Anderson KA, et al. Inclusion or exclusion? Exploring barriers to employment for low-income older adults. J Gerontol Soc Work. 2013;56(4):318–34. 10.1080/01634372.2013.777006. 23600601 10.1080/01634372.2013.777006

[20] Sen K, Prybutok V, Prybutok G. Determinants of social inclusion and their effect on the wellbeing of older adults. Innov Aging. 2020;4(Supplement_1):319–319. 10.1093/geroni/igaa057.1021.

[21] Carr DC, Fried LP, Rowe JW. Productivity & engagement in an aging America: the role of volunteerism. Daedalus. 2015;144(2):55–67. 10.1162/DAED_a_00330.

[22] Schwingel A, et al. Continued work employment and volunteerism and mental well-being of older adults: Singapore longitudinal ageing studies. Age Ageing. 2009;38(5):531–7. 10.1093/ageing/afp089. 19474036 10.1093/ageing/afp089

[23] Accius J, Suh JY. The Longevity Economy Outlook. Washington, DC: AARP Thought Leadership. 2020. Available at: 10.26419/int.00042.001.

[24] Scott AJ. The longevity society. Lancet Healthy Longev. 2021;2(12):e820–7. 10.1016/S2666-7568(21)00247-6. 36098038 10.1016/S2666-7568(21)00247-6

[25] Nguyen C et al. Adaptation for Growth Via Learning New Skills as a Means to Long-Term Functional Independence in Older Adulthood: Insights From Emerging Adulthood. The Gerontologist. 2018 [Preprint]. Available at: 10.1093/geront/gny128. 10.1093/geront/gny128 30321326

[26] Nielsen L, et al. New directions in geroscience: integrating social and behavioral drivers of biological aging. Psychosom Med. 2024;86(5):360–5. 10.1097/PSY.0000000000001320. 38718171 10.1097/PSY.0000000000001320 PMC12084117

[27] Thompson L. Training the new scientists of aging: building on geroscience opportunities. Innov Aging. 2023;7(Supplement_1):282–3. 10.1093/geroni/igad104.0940.

[28] Frow P, Payne A. A stakeholder perspective of the value proposition concept. Eur J Mark. 2011;45(1/2):223–40. 10.1108/03090561111095676.

[29] Schiller C, et al. A framework for stakeholder identification in concept mapping and health research: a novel process and its application to older adult mobility and the built environment. BMC Public Health. 2013;13(1):428. 10.1186/1471-2458-13-428. 23639179 10.1186/1471-2458-13-428 PMC3653754

[30] Vladimirova D. Building Sustainable Value Propositions for Multiple Stakeholders: A Practical Tool. 2019;7(1).

[31] Gariboldi MI, et al. Towards digital healthy ageing: the case of Agatha and priorities moving forward. The Lancet Regional Health - Western Pacific. 2023;35:100649. 10.1016/j.lanwpc.2022.100649. 37424690 10.1016/j.lanwpc.2022.100649 PMC10326683

[32] Singh S, Yadav R, Doskaliuk B. Aging and geriatric care: a global imperative towards universal health coverage. Anti-Aging Eastern Europe. 2023;2(2):76–81. 10.56543/aaeeu.2023.2.2.02.

[33] Gassmann O, Frankenberger K, Choudury M. The Business Model Navigator: 55+ models that will revolutionise your business. 2nd ed. New York: Pearson; 2020.

[34] Hsieh H-F, Shannon SE. Three approaches to qualitative content analysis. Qual Health Res. 2005;15(9):1277–88. 10.1177/1049732305276687. 16204405 10.1177/1049732305276687

[35] Freeman RE, editor. *Stakeholder theory: the state of the art*. 3. print. Cambridge: Cambridge Univ. Press; 2013.

[36] Giger O-F, et al. Collaboration and Innovation Patterns in Diabetes Ecosystems. 2024. Available at: 10.1101/2024.04.25.24306351.

[37] Hermes S, et al. The digital transformation of the healthcare industry: exploring the rise of emerging platform ecosystems and their influence on the role of patients. Bus Res. 2020;13(3):1033–69. 10.1007/s40685-020-00125-x.

[38] Retterath A, Braun R. Benchmarking Venture Capital Databases. Rochester, NY. 2020. Available at: 10.2139/ssrn.3706108.

[39] PitchBook. Venture Capital, Private Equity and M&A Database. 2024. Available at: https://pitchbook.com/. Accessed: 7 May 2024.

[40] Crunchbase. Crunchbase Inc. 2024. Available at: https://www.crunchbase.com/home. Accessed: 7 May 2024.

[41] Fried LP, Wong JE-L, Dzau V. A global roadmap to seize the opportunities of healthy longevity. Nature Aging. 2022:1–4. Available at: 10.1038/s43587-022-00332-7. 10.1038/s43587-022-00332-7 37118540

[42] Orduña-Malea E, Ayllón JM, Martín-Martín A, Delgado López-Cózar E. The lost academic home: institutional affiliation links in Google Scholar citations. Online Info Rev. 2017;41:762–81. 10.1108/OIR-10-2016-0302.

[43] Ebadi A, Schiffauerova A. How to boost scientific production? A statistical analysis of research funding and other influencing factors. Scientometrics. 2016;106(3):1093–116. 10.1007/s11192-015-1825-x.

[44] National Institute on Aging. Grants & Funding, National Institute on Aging. 2024. Available at: https://www.nia.nih.gov/research/grants-funding. Accessed: 2 December 2024.

[45] Rojas-Montesino E, et al. Analysis of scientometric indicators in publications associated with healthy aging in the world, period 2011–2020. Int J Environ Res Public Health. 2022;19(15):8988. 10.3390/ijerph19158988. 35897359 10.3390/ijerph19158988 PMC9329745

[46] Safavi K, et al. Top-funded digital health companies and their impact on high-burden, high-cost conditions. Health Aff. 2019;38(1):115–23. 10.1377/hlthaff.2018.05081. 10.1377/hlthaff.2018.05081 30615535

[47] Keller R, Hartmann S, Teepe GW, Lohse K-M, Alattas A, Car LT, et al. Digital behavior change interventions for the prevention and management of type 2 diabetes: systematic market analysis. J Med Internet Res. 2022;24(1):e33348. 10.2196/33348. 10.2196/33348 PMC8783286 34994693

[48] Mekniran W, Kowatsch T. Scalable Business Models in Digital Healthy Longevity: Lessons from Top-Funded Digital Health Companies in. 2023;2022:609–15. 10.5220/0011778400003414.

[49] Salamanca-Sanabria A, et al. Top-Funded Companies Offering Digital Health Interventions for the Prevention and Treatment of Depression: A Systematic Market Analysis. 2022. Available at: 10.2196/preprints.40754. 10.1186/s13690-024-01424-z PMC11533405 39497184

[50] Castro O, Salamanca-Sanabria A, Alattas A, Teepe GW, Leidenberger K, Fleisch E, et al. Top-funded companies offering digital health interventions for the prevention and treatment of depression: a systematic market analysis. Arch Public Health. 2024;82(1):200. 10.1186/s13690-024-01424-z. 10.1186/s13690-024-01424-z PMC11533405 39497184

[51] Dalle J-M, Besten M. den, Menon C. Using Crunchbase for economic and managerial research. Paris: OECD. 2017. Available at: 10.1787/6c418d60-en.

[52] Clarke V, Braun V, Hayfield N. Thematic analysis. Qualitative psychology: A practical guide to research methods. 2015;3:222–48.

[53] United Nations Department of Economic and Social Affairs. World Population Ageing 2020: Highlights: Living Arrangements of Older Persons, Statistical Papers - United Nations (Ser. A), Population and Vital Statistics Report. United Nations. 2021. 10.18356/9789210051934.

[54] Aboderin IAG, Beard JR. Older people’s health in sub-Saharan Africa. Lancet. 2015;385:e9–11. 10.1016/S0140-6736(14)61602-0. 25468150 10.1016/S0140-6736(14)61602-0

[55] Quashie N. Ageing and health in the Caribbean. Innov Aging. 2017;1:1258. 10.1093/geroni/igx004.4576.

[56] Stanley J, Chukwuorji J. Aging in sub-Saharan Africa. Innov Aging. 2024;8:523–4. 10.1093/geroni/igae098.1712. 10.1093/geroni/igae031 PMC11020234 38628824

[57] Balachandran A, de Beer J, James KS, van Wissen L, Janssen F. Comparison of population aging in Europe and Asia using a time-consistent and comparative aging measure. J Aging Health. 2020;32:340–51. 10.1177/0898264318824180. 30651037 10.1177/0898264318824180 PMC7322980

[58] Lima-Costa MF, de Andrade FB, de Souza PRB, Neri AL, Duarte YA de O, Castro-Costa E, de Oliveira C. The Brazilian Longitudinal Study of Aging (ELSI-Brazil): Objectives and Design. Am J Epidemiol. 2018;187:1345–1353. 10.1093/aje/kwx387. 10.1093/aje/kwx387 PMC6031009 29394304

[59] Bloom DE, Chatterji S, Kowal P, Lloyd-Sherlock P, McKee M, Rechel B, et al. Macroeconomic implications of population ageing and selected policy responses. Lancet. 2015;385:649–57. 10.1016/S0140-6736(14)61464-1. 25468167 10.1016/S0140-6736(14)61464-1 PMC4469267

[60] Babacan H. Public–Private partnerships for global health. In I. Kickbusch, D. Ganten, & M. Moeti (Eds.). Handbook of global health. Springer International Publishing; 2021. pp. 2755–2788.

