# Supplementary material for: The longevity landscape: mapping stakeholder priorities for healthy aging among high-income countries
Source: BMC Public Health. 2025 Dec 19;25:4267. doi: 10.1186/s12889-025-25498-8 (PMC12717712; doi:10.1186/s12889-025-25498-8)
Supplement: Supplementary file 1 — Supplementary Material 1. [file 12889_2025_25498_MOESM1_ESM.docx]

**Supplements**

**Table S1. Stakeholder descriptives**

| **Stakeholder Category** | **Organization Name** | **Country** | **Relevance Indicators** |
| --- | --- | --- | --- |
| Government agencies | National Institutes of Health USA | United States | 329 Publications |
| Government agencies | European Union | United Kingdom | 268 Publications |
| Government agencies | National Institute on Aging | United States | 182 Publications |
| Government agencies | National Institute for Health Research | United Kingdom | 55 Publications |
| Government agencies | United Nations | United States | 27 Publications |
| Government agencies | World Health Organization | Switzerland | 20 Publications |
| Government agencies | US National Academy of Medicine | United States | *2 Publications |
| Multilateral organizations | Wellcome Trust | United Kingdom | 113 Publications |
| Multilateral organizations | European Commission Joint Research Centre | Italy | 54 Publications |
| Multilateral organizations | Amsterdam Healthy Weight Approach (AHWA) | The Netherlands | *8 Publications |
| Multilateral organizations | AGE-WELL | Canada | *4 Publications |
| Multilateral organizations | Kaiser Permanente | United States | *3 Publications |
| Multilateral organizations | Gesundes Kinzigtal | Germany | *1 Publications |
| Multilateral organizations | Healthier SG | Singapore | *n/a |
| Multilateral organizations | International Institute on Ageing | Malta | *n/a |
| Multilateral organizations | National Council on Aging | United States | *n/a |
| Research centers | UK Research and Innovation | United Kingdom | 308 Publications |
| Research centers | Healthy Longevity Center at Uni Zurich, CH | Switzerland | *32 Publications |
| Research centers | Stanford University (Center on Longevity) | United States | *25 Publications |
| Research centers | Mayo Clinic | United States | *15 Publications |
| Research centers | National University of Singapore (Center for Healthy Longevity) | Singapore | 14 Publications |
| Research centers | Max Planck International Research Network on Aging | Germany | 13 Publications |
| Research centers | SENS Research Foundation | United States | *11 Publications |
| Research centers | Buck Institute for Research on Aging | United States | *8 Publications |
| Research centers | Massachusetts Institute of Technology (AgeLab) | United States | *7 Publications |
| Research centers | Chaim Sheba Medical Center | Israel | *4 Publications |
| Private companies | Altos Labs | United States | 3000000000 USD |
| Private companies | Devoted Health | United States | 2144000000 USD |
| Private companies | Hinge Health | United States | 2004655040 USD |
| Private companies | Grail | United States | 2004655040 USD |
| Private companies | WeDoctor | China | 1431500000 USD |
| Private companies | MGI Tech | China | 1200000000 USD |
| Private companies | Ping An Good Doctor | China | 1150000000 USD |
| Private companies | Miaoshou Doctor | China | 946669842 USD |
| Private companies | Lyra Health | United States | 910100000 USD |
| Private companies | HeartFlow | United States | 792722908 USD |
| Communities | Cedars Sinai | United States | 63310000 USD |
| Communities | MaineHealth | United States | 14149092 USD |
| Communities | Tipping Point Community | United States | 12000000 USD |
| Communities | VitaDAO | Canada | *11200000 USD |
| Communities | National Indian Health Board | United States | 10000000 USD |
| Communities | Association of Asian Pacific Community Health Organizations | United States | 9900000 USD |
| Communities | Heartland Family Service | United States | 5000000 USD |
| Communities | Catholic Charities Archdiocese of New Orleans | United States | 5000000 USD |
| Communities | Longevity DAO | United States | *n/a |
| Communities | Verein Lifegarten | Switzerland | *n/a |
| Opinion leaders | Aubrey de Grey | United States | 10 activity records |
| Opinion leaders | Dame Sarah Gilbert | United Kingdom | *4 activity records |
| Opinion leaders | Jim Mellon | Isle of Man | 3 activity records |
| Opinion leaders | S. Jay Olshansky | United States | 3 activity records |
| Opinion leaders | James Peyer | United States | 2 activity records |
| Opinion leaders | Anna King | United Kingdom | 2 activity records |
| Opinion leaders | Nathaniel David | United States | 2 activity records |
| Opinion leaders | Sergey Young | United Kingdom | 2 activity records |
| Opinion leaders | David Sinclair | Australia | *1 activity records |
| Opinion leaders | James Lovelock | United Kingdom | *1 activity records |

*Identified through purposeful sampling after a systematic search, where governmental agencies, multilateral organizations, and research centers were based on their respective relevance indicators, such as the number of associated publications, private companies, and local communities on the total funding amount, and opinion leaders on their reported number of activities.
